# Textured vs. Smooth Breast Implants Using the Jones Criteria—What Is the Currently Available Evidence for BIA-ALCL?: A Systematic Review

**DOI:** 10.3390/jpm13050816

**Published:** 2023-05-11

**Authors:** Andrzej Hecker, Barbara Giese, Anna-Lisa Pignet, Marlies Schellnegger, Lars-Peter Kamolz, David Benjamin Lumenta

**Affiliations:** 1 Research Unit for Digital Surgery, Division of Plastic, Aesthetic and Reconstructive Surgery, Department of Surgery, Medical University of Graz, 8036 Graz, Austria; 2 Division of Plastic, Aesthetic and Reconstructive Surgery, Department of Surgery, Medical University of Graz, 8036 Graz, Austria; 3 COREMED—Centre for Regenerative Medicine and Precision Medicine, Joanneum Research Forschungsgesellschaft mbH, 8010 Graz, Austria; 4 Medical University of Graz, 8036 Graz, Austria

**Keywords:** BIA-ALCL, smooth, textured, breast implants, jones classification

## Abstract

Breast-Implant-Associated Anaplastic Large-Cell Lymphoma (BIA-ALCL) is a rare low-incidence type of T-cell non-Hodgkin lymphoma, arising in the capsule around breast implants, and predominantly associated with the use of macro-textured breast implants. The purpose of this study was to use an evidence-based approach to systematically identify clinical studies comparing smooth and textured breast implants in women with regard to the risk of developing BIA-ALCL. Methods: A literature search in PubMed in April 2023 and the article reference list of the French National Agency of Medicine and Health Products decision from 2019 were screened for applicable studies. Only clinical studies where the Jones surface classification could be applied (required information: breast implant manufacturer) for comparison of smooth and textured breast implants were considered. Results: From a total of 224 studies, no articles were included due to the lack of fit to the strict inclusion criteria. Conclusions: Based on the scanned and included literature, implant surface types in relation to the incidence of BIA-ALCL were not evaluated in clinical studies and data from evidence-based clinical sources plays a minor to no role in this context. An international database that combines breast implant-related data from (national, opt-out) medical device registries is, therefore, the best available option to obtain relevant long-term breast implant surveillance data on BIA-ALCL.

## 1. Introduction

Breast-Implant-Associated Anaplastic Large-Cell Lymphoma (BIA-ALCL) is a rare form of T-cell non-Hodgkin lymphoma, which arises from the capsule around the surface of breast implants. Periprosthetic effusion, tissue formation, and other symptoms (e.g., unilateral breast enlargement, rash, and lymphadenopathy) can be associated with BIA-ALCL and may occur in the course of the disease. BIA-ALCL has a low incidence rate ranging from 1:3.817 to 1:30.000. So far, 1130 cases of BIA-ALCL, including 59 deaths, have been reported [1,2]. The development of BIA-ALCL seems to be multifactorial. On the one hand, evidence-based theories recognize genetic predisposition, the combination of chronic inflammation, and the time of tumorigenesis. On the other hand, research demonstrated an association between textured breast implants and the development of BIA-ALCL. Despite the available evidence from clinical cases, the underlying pathophysiological mechanism requires further scientific confirmation. The implant surfaces of breast implants have different manufacturing methods. Methods to produce a textured surface include salt loss, gas diffusion, imprint stamping, polyurethane foam coating, as well as other methods. The shell surface, also known as the outer layer, consists of silicone and different layers on top to increase their strength [3]. Texturing was introduced to improve the adhesion of the implants to the tissue and proclaimed to reduce the rate of capsular contracture. Several classifications to classify and compare breast implant surfaces exist. During the preparation of this work, no classification was found to be uniformly accepted [4]. Jones et al. proposed a new (texture) classification [5], which ranged from 1 to 4 (minimal, low, medium, and high risk) with an increased risk for developing BIA-ALCL in levels 3 (macro-textured breast implants) and 4 (polyurethane-coated breast implants). Most patients who developed BIA-ALCL had a textured breast implant. However, most reports do not provide precise and complete information about the surface texture of the implant at the time of diagnosis, and it was not possible to create a complete implant history of patients, especially in case of multiple revisions [6]. The French, followed by Irish authorities, introduced a ban on macro-textured and polyurethane-coated breast implants in April 2019. BIA-ALCL was introduced as a separate disease entity only in 2016 in the WHO classification, and reports remain rare on an international level. So far, no recommendation to retract textured implants from the market exists. Some professional societies still recommend the use of textured breast implants, while others restricted their use. Still, a trend to avoid the use of textured implants to reduce the risk of developing BIA-ALCL can be observed. Although no BIA-ALCL cases have been reported in smooth breast implants (without previous textured breast implants or textured expanders) to date, these observations, which have been made by scattered registries, need to be scientifically confirmed [2]. Based on this, we have opted for an evidence-based approach; we have chosen to conduct a systematic review that includes smooth breast implants as a reference group for textured breast implants. Therefore, this systematic review aimed to identify clinical evidence-based studies which compared smooth (Jones surface grade 1) and textured (Jones surface grade 2, 3, or 4) surface breast implants in women with regard to developing BIA-ALCL.

## 2. Materials and Methods

A systematic review of the scientific literature according to the Preferred Reporting Items for Systematic Reviews and Meta-Analysis (PRISMA) statement checklist [7]. The study was registered in the International Prospective Register of Systematic Reviews (PROSPERO) with the following registration number: CRD42020155937.

### Search Strategy and Article Selection

A literature search was performed in the online database PubMed using the following search term strategy: (“breast implant” OR “breast implants”) AND (“smooth” AND “textured”). The search was conducted in April 2023 for studies published in any year. Article reference lists and articles of the decision letter of the medical devices regulatory authority in France, the National Agency of Medicine and Health Products (ANSM), were also examined for applicable studies. Two authors (A.H. and B.G.) independently screened titles, abstracts, and, if available full articles identified in the online database PubMed. We applied a recently described surface area and roughness grading classification [5]. For an application of this Jones classification, breast implant manufacturer information is required. Only clinical breast implant studies that demonstrated a comparison of smooth (Jones surface grade 1) and textured (Jones surface grade 2, 3, or 4) breast implants with clinical outcomes between the surface types were considered. Articles including experimental studies involving laboratory studies and examinations on animals, reviews, commentaries or letters, breast implant surfaces not classified by Jones, and studies without BIA-ALCL as a primary endpoint were excluded. The reviewers (A.H. and B.G.) recorded all search results and available clinical-based data in an Excel sheet (Microsoft Excel 2016 Microsoft Office [16.44] 32-bit) independently and compared and reconciled their data. If a discrepancy occurred between the reviewers, the articles were evaluated by a third reviewer (D.B.L.).

## 3. Results

### Literature Search

**Figure 1:**
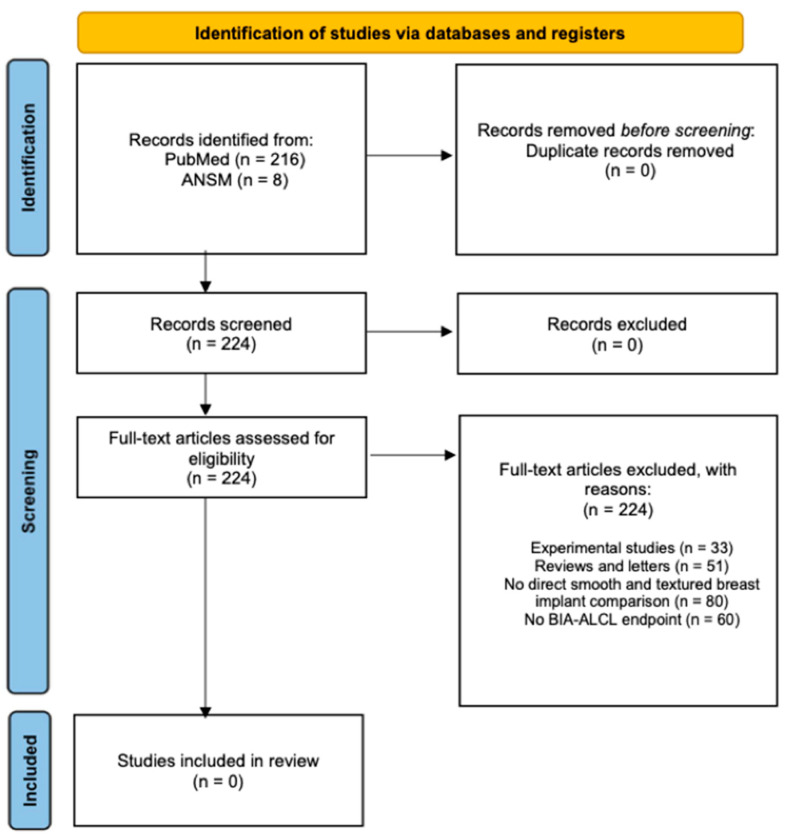
PRISMA Flow Diagram. PRISMA: Preferred Reporting Items for Systematic Reviews and Meta-Analyses; ANSM: National Agency of Medicine and Health Product in France.

The initial literature search yielded 216 studies in PubMed and 8 of the ANSM reference list. Ultimately, 224 studies out of the total 224 studies were excluded. Articles were excluded according to the prior mentioned exclusion criteria, including experimental studies (n = 33), reviews, letters or commentaries (n = 51), no comparison between smooth and textured implant surface types (n = 80), no BIA-ALCL as a primary endpoint (n = 60), and where the Jones criteria could be applied (required information: implant manufacturer) (n = 0).) After applying our exclusion criteria, no clinical studies have been found to demonstrate a comparison of smooth (Jones surface grade 1) and textured (Jones surface grade 2, 3, or 4) breast implants with clinical outcomes with regard to developing BIA-ALCL. No study was included in the analysis (Figure 1).

## 4. Discussion

The following chapter aims to give an overview of the possible consequences of a textured breast implant ban, current challenges, and an approach to obtaining evidence-based data for rare diseases.

### 4.1. Ban of Textured Breast Implants: An Adequate Precautionary Consequence?

BIA-ALCL is a rare disease with a relatively good prognosis if detected at an early disease stage. Some board-certified societies have no recommendation for/against textured breast implants; other countries altogether banned their use [8]. Moreover, some have begun avoiding the use of textured implants to reduce the risk for developing BIA-ALCL [9]. Considering the international controversy over the use of textured breast implants, a comparison between the benefits of smooth and textured breast implants showed several clinical benefits in textured breast implants [10]. These include not only reduced capsular contracture and reduced implant displacement but also reduced stretch in the lower pole of the breast resulting in reduced device rotation over time [10]. A cost-effectiveness analysis of smooth breast implants compared with textured breast implants for breast augmentation surgery demonstrated that the implantation of smooth breast implants resulted in higher social costs in relation to the individual than the use of textured ones. The incremental cost for breast augmentation with smooth breast implants was USD 1469 per patient [9]. They argued that further reoperations could not be prevented by the increased use of smooth breast implants. A total of 2925.65 additional reoperations for utilizing smooth breast implants over textured ones had to be performed just to prevent one case of BIA-ALCL [9]. However, an exact manufacturer-specific risk is still not known [10,11,12,13]. In the absence of sufficient breast implant-based BIA-ALCL data, the recommendation to remove all textured implants from the market may be overcautious. Decisions on whether to ban any type of implant should be determined via evaluation of the risks and benefits, using complete numerator and denominator data and the application of standardized breast implant surface classifications.

### 4.2. Limitations to Obtaining Evidence-Based BIA-ALCL Data

#### 4.2.1. Incomplete Numerator and Denominator Data

Due to the lack of adequately run international breast implant registries, the total number of women with breast implants remains unknown. When the first case of BIA-ALCL was reported in 1997 by Keech and Creech [14], BIA-ALCL was finally declared by the Word Health Organization (WHO) in 2016 [15] as its own new disease entity. Since only 1130 cases have been reported internationally so far and the average duration of development after implant placement can take up to 7 to 32 years [16], conducting clinical trials is hardly possible for rare illnesses such as BIA-ALCL. For an adequate risk calculation, the total number of women with BIA-ALCL (numerator) and the total number of women with breast implants (denominator) is required. The only recent WHO declaration and the late onset of the disease may be directly related to an underreporting of BIA-ALCL. With incomplete numerator and denominator data, clinical trials or (systematic) reviews, such as this one, represent an insufficient approach to contribute to the evidence of BIA-ALCL.

#### 4.2.2. Absence of Standardized Breast Implant Surface Classification

Among the internationally available breast implant surface classifications, to the best of our knowledge, the Jones Classification is the only scientifically published one that compares the different texturing methods of the manufacturers resulting in different categories of surface roughness and divides the risk from 1 to 4 (minimal, low, medium, and high risk) [5]. These categories, based on the manufacturer, support a re-evaluation of textured breast implants as a spectrum of medical devices with a surface-based variable risk. Provided that manufacturer information is available, the Jones classification can be retrospectively applied. The Jones classification of breast implant manufacturers regarding the different categories of surface roughness is shown in Table 1. Additionally, several different classifications to compare the surfaces of breast implants have become available [17]. These classification systems vary in their criteria, such as surface area, tissue adherence, capsule formation, hydrophobicity, bacterial adherence, and fibroblast and macrophage activity [4]. While the ISO (International Organization for Standardization) currently categorizes texturization grade into smooth, microtextured, and macro-textured categories, the Jones classification also incorporates bacterial adherence in their grading scheme, which plays a possible role in breast implant-related complications (e.g., capsular contracture, BIA-ALCL) [4,5]. For that reason, we have opted for this recent classification. Just like the Jones classification, each classification system assigns breast implant manufacturers according to their criteria. However, none of these classification systems has been clinically validated in a prospective manner. The lack of standardization significantly limits the scope of our as well as other types of systematic analyses. The application of standardized surface classifications is only possible as soon as breast implant data have become available. Therefore, we recommend reporting the following breast implant-related information concerning breast implant-related conditions (e.g., BIA-ALCL, capsular fibrosis) in articles for future reviews: manufacturer, breast implant shell texture, implant covering and shape, type of implant fill, breast implant history (e.g., years since implantation, multiple device exposure, history of prior tissue expander).

### 4.3. Approach to Obtaining Evidence-Based BIA-ALCL Data

#### 4.3.1. International BIA-ALCL Register: Combination of Medical Device Data and Clinical Data

The exchange of medical devices and clinical data still remains a globally recognized challenge in clinical data science. The majority of countries worldwide still have not developed a concomitant registration of breast implants and the associated development of BIA-ALCL [18,19,20,21]. Successful data collection requires ethical approval and consent processes accounting for an appropriate privacy and legislative framework. In addition, the complexity of the data collection and the associated compliance in international exchange represents a potential barrier [22].

The application of the FAIR Data Principles (Findable—Accessible—Interoperable, and Reusable) can enable worldwide comparability of medical datasets. Therefore, the following prerequisites are required for the establishment of a successfully and globally standardized breast implant registry: The first principle, “Findable”, describes the unique patient identifier (UPI), including the age of the patient, patient records, definitions of patient’s state as well as their consent. The voluntary participation of breast implant holders in a registry revealed an insufficient dataset (opt-in). Instead, the term “opt-out” was introduced to represent institutional permission to enroll all patients automatically onto a registry unless they opt out, giving an appropriate statement. Additionally, an unique device identifier (UDI) of the breast implant retracting to the providers, manufacturing procedures, lacks, or malfunctions are needed. Another essential step for the detection of rare diseases is the monitoring of clinical aspects, which includes diagnostic, therapeutic measures, and clinical endpoints [22,23].

The requirement for global interaction is the international retrieval of medical data, respecting privacy requirements (“Accessible”). “Interoperable” represents a standardized, globally accessible, and common language that should apply the FAIR principles. The principle of “reusable” includes the description of data with relevant attributes to finally differentiate or even extend the medical data set with other available ones. These principles are reflected in the International Collaboration of Breast Registry Activities (ICOBRA) [23,24]. Other registry efforts include the “Patient Registry and Outcomes for Breast Implants and Anaplastic Large Cell Lymphoma Etiology and Epidemiology” (PROFILE registry) [25] and other nationally driven BIA-ALCL registrations [26,27,28]. An international registry database that collects and unifies clinical and breast implant-related data currently appears to be the best way to obtain sufficient data for further risk assessment of BIA-ALCL.

#### 4.3.2. BIA-ALCL Registry: A Possibility for Personalized Medicine

The personalization of diagnostic and therapeutic measures is one of the major factors in improving individual outcomes [24,25]. BIA-ALCL registries can enable increased patient safety through a standardized and continuous collection of medical device and clinical data from the individual [23]. This approach includes the exact monitoring of breast implants. In combination with clinical datasets of each patient with a breast implant, a global early warning system can be created. Additionally, standardized diagnostic and therapeutic measures facilitate the diagnosis and improve the prognosis of this rare disease [19]. Complete and combined data in a standardized BIA-ALCL registry could finally lead to a better understanding of the multifactorial pathogenesis of BIA-ALCL.

## 5. Conclusions

The lack of clinical data, the rarity of the disease, its long-term duration until presentation following implantation, as well as the non-standardized collection of breast implant surface data, resulted in insufficient data for elaborate research evaluation. Thus, we found no literature-based evidence. A complete long-term data set based on above mentioned principles from international collaborations can ultimately allow us to weigh the risk and benefits of breast implants and their related surface types and aid in developing a more personalized approach, when it comes to breast implant selection. According to that, data from evidence-based clinical sources plays a minor to no role in this context. An international database that combines breast implant-related data from (national, opt-out) medical device registries is, therefore, the best available option to obtain relevant long-term breast implant surveillance data on BIA-ALCL.

## 6. Limitations

Within this systematic review, we limited our search to the PubMed database and the ANSM reference list. We used a non-specific search string ((“breast implant” OR “breast implants”) AND (“smooth” AND “textured”)) to detect and screen all potential implant-related studies, resulting in a non-negligible number of studies which had to be manually filtered and ultimately excluded based on our criteria.

## Figures and Tables

**Table 1:** Overview of Jones classification [4,5]. PU: polyurethane foam.

Classification	Texturing Method	Manufacturer
1 Minimal	/	All smooth
		Motiva Silk/Velvet
2 Low	Imprinting Salt loss	Mentor Siltex
		Nagor
3 Intermediate	Salt loss	Allergan Biocell
		Eurosilicone
4 High	Polyurethane bonded foam	Polytech PU
		Surgitek PU Silimed PU

## Data Availability

Detailed data supporting the results are available from the authors.
